# Efficacy and safety of electroacupuncture for post stroke depression: study protocol for a randomized controlled trial

**DOI:** 10.1186/s13063-018-2548-0

**Published:** 2018-03-02

**Authors:** Wa Cai, Wen Ma, Guan-Tao Wang, Wei-Dong Shen

**Affiliations:** Department of Acupuncture, Shanghai Shuguang Hospital, Shanghai University of Traditional Chinese Medicine, Shanghai, 201203 China

**Keywords:** Electroacupuncture, Poststroke depression, Randomized controlled trial

## Abstract

**Background:**

Poststroke depression is closely related to increased mortality in stroke patients. Compared with antidepressants, electroacupuncture (EA) treatment for poststroke depression (PSD) has relatively more stable effectiveness and can reduce side effects. This trial is designed to provide solid evidence for the efficacy and safety of EA treatment for patients with PSD.

**Methods/design:**

This ongoing study is a single-blind, single-center, parallel group, randomized controlled trial. Sixty-two participants will be recruited from Shanghai Shuguang Hospital and randomized into either the EA group or the sham EA group. Baihui, Sishencong, Ganshu, Sanyinjiao, and Taichong are selected as the treatment acupoints in both groups. The EA group will receive the traditional EA treatment with de-qi sensation, and the sham EA group will receive sham EA treatment without needle penetration and electrostimulation. Participants will receive treatment 3 times per week for a total of 12 sessions over 4 weeks. The primary outcome is Hamilton Rating Scale for Depression score, and the secondary outcomes are scores on the Zung Self-Rating Depression Scale, National Institutes of Health Stroke Scale, Barthel Index of Activities of Daily Living, and Depression Scale of traditional Chinese medicine. All of the outcome measures will be assessed at baseline, 2 weeks after EA treatment onset, 4 weeks after treatment onset, and at 8-week follow-up. Safety assessments will be done at each visit.

**Discussion:**

The results of this trial will demonstrate the efficacy and safety of EA treatment for PSD with credible and important clinical evidence, thus supporting EA treatment as an ideal choice for PSD treatment.

**Trial registration:**

Chinese Clinical Trial Registry, ChiCTR-IOR-17012610. Registered on 7 September 2017. http://www.chictr.org.cn/edit.aspx?pid=21494&htm=4

**Electronic supplementary material:**

The online version of this article (10.1186/s13063-018-2548-0) contains supplementary material, which is available to authorized users.

## Background

Stroke, a cardiovascular disorder with relatively high mortality, is a leading cause of serious long-term disability [1]. Researchers have shown that several neuropsychiatric problems, including depression, anxiety, dementia, apathy, and psychosis, may occur after stroke. Statistically, symptoms of depression occur in one-third of stroke survivors [2]. Thus, poststroke depression (PSD) is the most frequent neuropsychiatric sequela of stroke. PSD, whose major symptoms are melancholia and dysphoria, does harm to cognitive function, social activity, and stroke rehabilitation, which is closely related to the increase of mortality [3].

In the treatment of PSD, antidepressants such as citalopram, escitalopram, nortriptyline, milnacipran, mirtazapine, piracetam, and fluoxetine are most commonly used by Western doctors nowadays. However, it has been widely verified that the use of antidepressants in patients with PSD has an increased risk of adverse events (AEs) that may lead to other problems, including blurry vision, urinary retention, hypotension, sexual dysfunction, tremor, and severe insomnia [4]. Psychotherapy interventions, including cognitive behavioral therapy [5], a social work intervention using problem solving [6], advice on services and counseling, motivational interviewing [7], and a supportive intervention [8], have been demonstrated to be effective for the treatment of PSD [4], but currently the literature regarding the efficacy of psychological therapy for PSD is limited. The use of electroconvulsive therapy (ECT), involving the electrical induction of seizures in patients, is an effective protocol for treatment of PSD [9]. Nevertheless, some severe side effects of ECT, such as confusion, headache, nausea [10], mania, cognitive dysfunction, prolonged seizures, prolonged apnea, hypertension, and arrhythmia [11], have also been reported.

Acupuncture, a traditional Chinese therapy using needles to puncture into acupoints on the body surface, is based on the theory of meridians and the balance of yin, yang, qi, and blood [12, 13]. Currently, a number of clinical reports have demonstrated that electroacupuncture (EA) treatment for PSD is significantly effective with few side effects, but there are few randomized controlled trials (RCTs) showing this [14, 15]. Hence, lack of standard clinical RCTs results in insufficient evidence to support EA treatment for PSD. It is necessary to design a high-quality clinical RCT for PSD treated by EA. Our proposed trial will be conducted as a parallel group RCT to comprehensively evaluate the efficacy and safety of EA treatment for PSD by comparing an EA group with a sham EA group.

## Methods/design

### Hypotheses

1. Patients with PSD with 12 sessions (4 weeks) of EA treatment will have certain improvements in their symptoms and QoL scores.
2. Patients with PSD treated with EA will experience alleviation of their symptoms and have increased QoL scores significantly greater than those treated by sham EA.

### Study design

**Fig. 1:**
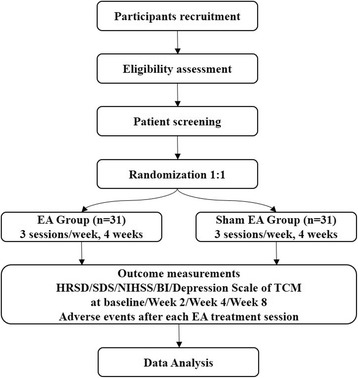
The planned flowchart of the trial. *BI* Barthel Index of Activities of Daily Living, *EA* Electroacupuncture, *HRSD* Hamilton Rating Scale for Depression, *NIHSS* National Institutes of Health Stroke Scale, *SDS* Zung Self-Rating Depression Scale, *TCM* Traditional Chinese medicine

**Fig. 2:**
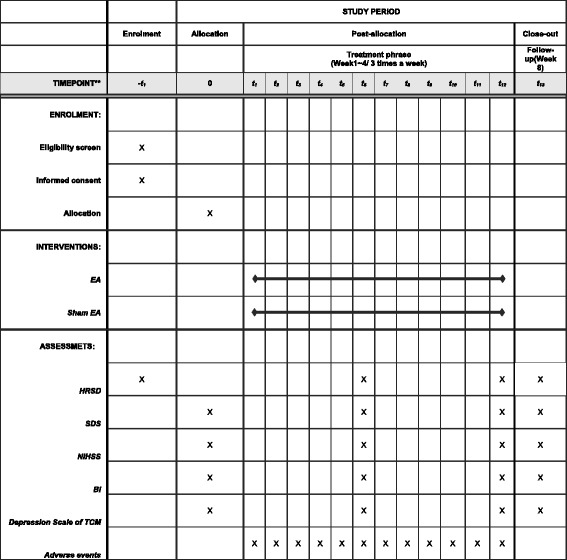
The study schedule for enrollment, treatments, outcome measurements and data collection. *EA* Electroacupuncture, *HRSD* Hamilton Rating Scale for Depression, *SDS* Zung Self-Rating Depression Scale, *NIHSS* National Institutes of Health Stroke Scale, *BI* Barthel Index of Activities of Daily Living, *TCM* Traditional Chinese medicine

We plan to conduct a single-center, patient-blinded, assessor-blinded, parallel group RCT strictly in accordance with the Consolidated Standards of Reporting Trials (CONSORT) [16] and STandards for Reporting Interventions in Clinical Trials of Acupuncture (STRICTA) guidelines [17]. The study will be conducted from October 2017 to October 2019 at Shuguang Hospital, affiliated with Shanghai University of Traditional Chinese Medicine in Shanghai, China. Figure 1 shows the planned flowchart of the trial. Figure 2 demonstrates the study schedule for enrollment, treatments, outcome measurements, and data collection. Additional file 1 contains the Standard Protocol Items: Recommendations for Interventional Trials (SPIRIT) checklist.

### Participants

#### Patient screening

The following patient screening will be done to conduct a safety assessment of patients and exclude patients with fatal conditions such as coma, complete paralysis, secondary intracerebral hemorrhage, and severe infections resulting from stroke:

1. History inquiry and recording
2. Physical examination, including muscle strength test, physiological reflex, pathological reflex, and Brunnstrom movement function assessment and coordination movement assessment
3. Laboratory tests, including complete blood count, C-reactive protein, and urinalysis
4. Brain computed tomography

#### Inclusion criteria

1. Patients aged 40–85 years with no sex restriction
2. Patients who meet the diagnostic criteria of the guidelines for the diagnosis and treatment of patients with acute ischemic stroke [18] and the diagnostic criteria of insomnia according to the *Diagnostic and Statistical Manual of Mental Disorders, Fifth Edition* [19]
3. Patients whose depression symptoms occur after stroke and whose Hamilton Rating Scale for Depression (HRSD) scores are between 8 and 35
4. Patients willing to participate in the RCT and sign informed consent forms

#### Exclusion criteria

1. Patients in a state of coma or with severe disturbance of consciousness, aphasia, agnosia, or deafness that subsequently affects expression and communication
2. Patients with complications of severe heart failure, respiratory failure, renal insufficiency, other severe diseases in acute stage, or other neurological/musculoskeletal disorders that affect functional rehabilitation
3. Patients who are afraid of EA treatment or are reluctant to accept it

#### Dropout criteria

1. Patients who accept other treatments except EA for PSD during observation period
2. Patients who fail to or are unwilling to finish the EA treatment schedule

#### Recruitment strategies

Several strategies will be applied to participant recruitment. In this RCT, we will recruit mainly participants who are hospitalized patients at acupuncture departments and neurology departments in Shanghai Shuguang Hospital. We will put up posters in the hospitals and advertisements across WeChat (a Chinese multipurpose social media mobile application software), which will give brief descriptions of the population needed to be enrolled (with 62 being the goal population for this trial); the free acupuncture treatments offered to participants; the purpose, procedures, treatments, and possible risks of the trial; and the contact information of the researchers. After the screening test, all of the participants will be required to sign the consent forms and assigned to different intervention groups by another researcher afterward. Clinical recruitment staff will be responsible for the recruitment process.

### Randomization and allocation

The block randomization method will be used by a specified researcher who is not involved in the trial. Randomization is planned to be conducted by using IBM SPSS Statistics version 24.0 software (IBM, Armonk, NY, USA) to generate a random number table that allocates the eligible participants to either of two groups (the EA group and the sham EA group) in a 1:1 ratio after they have signed printed informed consent forms. The random allocation cards will be made and sealed in opaque envelopes. Patients will be assigned into different groups by another researcher who is also responsible for informing the acupuncturists of the group allocation.

### Blinding

Because this is a single-blind study, participants, outcome assessors, and data statisticians will be blinded to the group allocation. However, acupuncturists will not be blinded, because sham acupuncture is definitely practiced differently from real acupuncture. Each participant will be assigned to receive EA treatment at different times in separate treatment rooms to avoid communication, and acupuncturists will have been trained previously not to inform other researchers, including outcome assessors and data statisticians as well as the participants, about group allocation information. Blinding will be maintained during the whole study process. James et al.’s blinding index will be evaluated after the completion of the study to evaluate the success of blinding [20].

### Interventions

All the eligible participants will be randomized into one of two groups: the EA group or the sham EA group. The EA treatment, consisting of 12 sessions across 4 weeks with 3 sessions each week, will be performed by experienced acupuncturists to ensure identical acupuncture treatment according to standard operating procedures. The participants in both groups will receive treatment by acupuncturists at the same acupoints as follows: Baihui (DU20), Sishencong (EX-HN1), Ganshu (BL18), Sanyinjiao (SP6), and Taichong (LR3), all of which are the most frequently used acupoints for treating PSD according to previous research. The needle retention time in both groups is 30 minutes. In addition to EA treatment, the participants will receive basic Western medical therapy for stroke (Table 1).

**Table 1:** Intervention details of both groups

	EA group	Sham EA group
Acupoints	DU20, EX-HN1 BL18, SP6, LR3	DU20, EX-HN1 BL18, SP6, LR3
Needle penetration	DU20, EX-HN1: 10 mm BL18, SP6, LR3: 15 mm	No needle penetration
Needle stimulation	De-qi sensation	No de-qi sensation
Electrostimulation	Current frequency: 2/100 Hz	No electrostimulation
Needle retention time	30 minutes	30 minutes
Treatment tools	Stainless steel needles and a PG-306 electroacupuncture device	A Park Sham Device with a PG-306 electric pulse generator with a cut wire inside
Treatment frequency and duration	Three sessions per week, 4 weeks	Three sessions per week, 4 weeks

*Abbreviations: BL18* Ganshu, *DU20* Baihui, *EA*, Electroacupuncture, *EX-HN1* Sishencong, *LR3* Taichong, *SP6* Sanyinjiao

#### Electroacupuncture group

Stainless steel needles and a PG-306 EA device will be used in treating with participants in the EA group. Acupuncturists will sterilize participants’ skin and then puncture DU20 and EX-HN1 horizontally 10 mm into the scalp skin. SP6 and LR3 will be punctured vertically 15 mm into the skin, and BL18 will be punctured obliquely 15 mm into the skin. Needling sensation (De-qi sensation) will be obtained through needle manipulation, including lifting, thrusting, and rotating. The needles will be stimulated using an electric current at a frequency of 2/100 Hz with a PG-306 EA device for about 30 minutes per EA treatment session.

#### Sham electroacupuncture group

A Park Sham Device with blunted needles that do not penetrate the skin, as described previously [21–23], will be applied to the participants in the sham EA group. The sham device, installed with a PG-306 electric pulse generator with a cut wire inside, will give only a little stimulation but a fake sound resembling the electrostimulation. Thus, there is no needle penetration and no electric current stimulation through the use of the Park Sham Device.

### Outcomes

An independent researcher who is blinded to the group allocation will be in charge of the outcome assessment.

#### Primary outcome

The changes in HRSD scores between baseline, 2 weeks after EA treatment onset, 4 weeks after treatment onset, and at 8-week follow-up will be set as the primary outcome of the clinical study.

##### Hamilton Rating Scale for Depression

The HRSD, originally published by Max Hamilton [24], is a multiple-item questionnaire that can provide an indication of depression and act as a guide for evaluating recovery. It is designed to assess depression severity by probing mood, feelings of guilt, suicidal ideation, insomnia, agitation or retardation, anxiety, weight loss, and somatic symptoms. The participants will be rated along 24 dimensions with scoring on a 3- or 5-point scale. A score of 0–7 is considered to be normal, and scores of 20 or higher indicate moderate, severe, or very severe depression. The assessment time is around 20 minutes [25]. The HRSD, evaluated by the researcher to assess the degree of depression of patients with PSD, can give an objective and reflective outcome.

#### Secondary outcomes

Secondary outcomes include the Zung Self-Rating Depression Scale (SDS), National Institutes of Health Stroke Scale (NIHSS), Barthel Index of Activities of Daily Living (BI), and Depression Scale of traditional Chinese medicine (Depression Scale of TCM), all of which will be evaluated at the same time points as the assessment of the primary outcome.

##### Zung Self-Rating Depression Scale

Designed by Duke University psychiatrist William W. K. Zung [26], the SDS is intended to assess the level of depression of patients who have been diagnosed with depressive disorder. A short self-rated questionnaire, it consists of 30 items that rate the affective, psychological, and somatic symptoms closely related to depression. Each question is scored on a scale of 1 through 4 based on responses “*a little of the time,” “some of the time,” “good part of the time,” and “most of the time*.” Scores fall into four ranges as follows: 20–44 indicates normal, 45–59 indicates mildly depressed, 60–69 manifests moderately depressed, and 70 and above defines severely depressed [27]. The SDS, assessed by patients with PSD themselves, can present a subjective and direct outcome.

##### National Institutes of Health Stroke Scale

The NIHSS, including domains such as level of consciousness, eye movements, visual fields, facial movements, muscle strength, sensation, coordination, language, speech, and neglect, is a 15-item scale used to measure neurological deficits and impairments caused by stroke. Each impairment is scored on a scale ranging from 0 to 2, 0 to 3, or 0 to 4. Scores are summed to range from 0 to 42. The higher the score, the more severe the neurological deficit [28].

##### Barthel Index of Activities of Daily Living

The BI is a scale considered to measure performance in activities of daily living, and each item is rated on the scale with a given number of points for each level [29]. Ten variables are used to describe activities of daily living and mobility. The BI has been used mainly to monitor functional outcomes related to stroke. A higher number indicates a greater likelihood of stroke patients living at home with a degree of independence after leaving the hospital.

##### Depression Scale of traditional Chinese medicine

To evaluate the degree of liver qi stagnation in the participants, the Depression Scale of TCM consists of six items concerning symptoms such as chest distress, belching, palpitation, insomnia, fatigue, irritability, and weeping. Each item is scored on a scale ranging from 0 to 3. Lower score sums manifest a slighter degree of liver qi stagnation. The Depression Scale of TCM, intended to reveal the exact TCM syndrome of depression, can be used to evaluate the EA treatment outcome of PSD from the perspective of TCM.

### Safety assessment

Participants’ safety will be evaluated during the whole EA treatment to avoid AEs, including local bleeding or pain at the acupuncture points, local redness or bruising, itching, and dizziness during treatment. At each visit, the time of occurrence, severity, progress, and treatment of AEs will be recorded in detail. If any serious AE occurs, the principal investigator and the institutional review board will be informed immediately, and direct actions will be taken right away.

### Sample size

According to the difference of HRSD scores between the two groups, the required sample size of each group will be carefully calculated by the data statistician. Based on our previous pilot study, the change in the SD of HRSD scores in the sham EA group is 5 points and that in the EA group is 13 points. If the HRSD score change in the EA group is 8.5 points higher than that in the sham EA group, it will be considered clinically significant. On the basis of a 5% false-positive error rate (α = 0.05, two-sided) and 80% power (β = 0.2), the sample size is estimated as follows:

$$ {\displaystyle \begin{array}{l}n=\frac{{\left({Z}_{1-\alpha }+{Z}_{1-\beta}\right)}^2\times \left({\sigma}_1^2+{\sigma}_2^2\right)}{\delta^2}\\ {}\kern1.5em =\frac{{\left(1.96+1.28\right)}^2\times \left({5}^2+{13}^2\right)}{8.5^2}\approx 28.48\end{array}} $$

A sample size of at least 29 participants should be recruited in each group. Assuming about a 10% dropout rate, which has been verified in the pilot study, a total of 62 participants will be enrolled in the trial.

### Data collection, management, and monitoring

The case report form and adverse events form will be carefully checked and input into the computer by two independent researchers to ensure data accuracy. All the original forms will be archived in the clinical research center of Shanghai Shuguang Hospital. The study will be monitored monthly by the data and safety monitoring board (DSMB) of the clinical evaluation center of Shanghai Shuguang Hospital, which will make the final decision to terminate the trial if any serious acupuncture-related AE occurs. The data manager of the DSMB can have access to the final dataset and has the right to give approval to others who need to gain access to the final dataset.

### Statistical analysis

An independent statistician will use IBM SPSS Statistics 24.0 software to conduct the whole data analysis. According to the intention-to-treat (ITT) principle, both ITT and per-protocol (PP) analyses will be performed. Any participant who receives treatment more than nine times during the study will be enrolled in the PP analysis. In terms of ITT analysis of secondary outcomes, the last observation carried forward rule will be used as the strategy in management of missing data. For the primary outcome, the maximum likelihood estimation method will be used.

All continuous variables will be presented as mean ± SD, and categorical variables will be presented as number (percent). The statistical significance level is considered to be 0.05 (two-sided) with 95% CIs. The homogeneity of the baseline characteristics between the two groups is designed to be tested by the two-sample *t* test for quantitative data and the chi-square test for qualitative data. If there is a possibility of covariance, analysis of covariance (ANCOVA) will be used for adjustment of baseline characteristics. To compare variables between the two groups, the parametric two-sample *t* test for normally distributed data and the nonparametric Mann-Whitney *U* test for nonnormally distributed data will be used. Repeated-measures ANCOVA will be applied to analyze different time point assessments between groups and interaction between groups and observed time. Differences within groups will be assessed with the paired *t* test (parametric method) and the Wilcoxon signed-rank test (nonparametric method).

### Quality control

To ensure the quality of this trial, the monitoring, consisting of case report forms, participants’ compliance with EA treatments, a trial master file, serious AE records, and dataset, will regularly and strictly be done by the DSMB of the clinical evaluation center of Shanghai Shuguang Hospital.

### Clinical trial registration

To reduce bias, increase transparency, and guarantee the high quality of the trial, this RCT has registered with the Chinese Clinical Trial Registry (ChiCTR-IOR-17012610).

## Discussion

Previous trials have verified the effect of acupuncture on alleviating PSD. However, most of those trials were designed to compare the therapeutic effect between an acupuncture group and a Western medicine group or between groups treated with acupuncture of different types [30–34]. Thus, it is difficult to assess the specific effect of acupuncture rather than its placebo effect. Li et al. considered the placebo effect of acupuncture and compared an acupuncture plus oral placebo group with nonacupoint spots with a shallow needling plus oral fluoxetine group [35]. Man et al. compared a group that received dense cranial EA stimulation plus selective serotonin reuptake inhibitors (SSRIs) plus body EA with a group treated with noninvasive cranial EA plus SSRI plus body EA [15]. However, accurate assessment of the specific effect of acupuncture is hard to realize in this way because of the combined interventions (acupuncture plus Western medicine) instead of a single intervention (acupuncture). Thus, our present RCT has been designed to use a sham acupuncture method that is based on a Park Sham Device installed with a PG-306 electric pulse generator by which needle penetration and electrostimulation are completely avoided, with the intent being to successfully prevent participants from knowing what their exact treatment is and to which group they belong. Only in this way can the disturbance of acupuncture treatment’s placebo effect or patients’ psychological factors be effectively reduced.

In terms of the selected acupoints, DU20 and EX-HN1 are located on the top of head, stimulation of which is able to induce resuscitation of patients with PSD. The combination of SP6, LR3, and BL18 leads to significant alleviation of symptoms of depression by dispersing liver qi and relieving qi stagnation that is considered to be the leading TCM pathogenesis of PSD according to TCM theory. Considering the previous studies on acupuncture treatment for PSD, the acupoints selected in this trial are the most frequently used by clinical practitioners and researchers and turn out to be more effective than other acupoints.

Nevertheless, there are some limitations of this trial. It is impossible to blind the acupuncturist who is responsible for treating patients from different groups with different EA methods, so it is rather hard to make the RCT double-blinded. This trial is also restricted to a single center. Furthermore, though the use of a Park Sham Device can give patients the fake sense of needle puncture and hearing the fake sound of electrostimulation, it is also difficult to ensure sham control effects by objective methods or quantitative criteria.

In conclusion, this RCT, following the CONSORT [16] and STRICTA [17] guidelines, can meet the needs of evaluating the efficacy and safety of EA treatment for patients with PSD and providing neurologists and psychologists or researchers from related fields with more solid evidence for TCM practitioners to give EA treatment for PSD. It is expected that our further study of EA treatment for PSD will be conducted in multicentered hospitals with an expanded sample size based on the experiences in the present study.

## Trial status

Participants are being recruited. The trial is planned to be completed by 30 October 2019.

## Additional file

Additional file 1: SPIRIT checklist. (DOCX 22 kb)

## Abbreviations

AE: Adverse event
ANCOVA: Analysis of covariance
BI: Barthel Index of Activities of Daily Living
BL18: Ganshu
CONSORT: Consolidated Standards of Reporting Trials
DSMB: Data and safety monitoring board
DU20: Baihui
EA: Electroacupuncture
ECT: Electroconvulsive therapy
EX-HN1: Sishencong
HRSD: Hamilton Rating Scale for Depression
ITT: Intention to treat
LR3: Taichong
NIHSS: National Institutes of Health Stroke Scale
PP: Per protocol
PSD: Poststroke depression
RCT: Randomized controlled trial
SDS: Zung Self-Rating Depression Scale
SP6: Sanyinjiao
SPIRIT: Standard Protocol Items: Recommendations for Interventional Trials
SSRI: Selective serotonin reuptake inhibitor
STRICTA: STandards for Reporting Interventions in Clinical Trials of Acupuncture
TCM: Traditional Chinese medicine

## References

[1] Go AS, Mozaffarian D, Roger VL, Benjamin EJ, Berry JD (2013). Heart disease and stroke statistics—2013 update: a report from the American Heart Association. Circulation.

[2] Hackett ML, Pickles K (2014). Part I: frequency of depression after stroke: an updated systematic review and meta-analysis of observational studies. Int J Stroke.

[3] Ellis C, Zhao Y, Egede LE (2010). Depression and increased risk of death in adults with stroke. J Psychosom Res.

[4] Hackett ML, Anderson CS, Xia J, House A (2008). Interventions for treating depression after stroke. Cochrane Database Syst Rev.

[5] Lincoln NB, Flannaghan T (2003). Cognitive behavioral psychotherapy for depression following stroke: a randomized controlled trial. Stroke.

[6] Towle D, Lincoln NB, Mayfield LM (1989). Service provision and functional independence in depressed stroke patients and the effect of social work intervention on these. J Neurol Neurosurg Psychiatry.

[7] Watkins CL, Auton MF, Deans CF, Dickinson HA, Jack CI, Lightbody CE, Sutton CJ, van den Broek MD, Leathley MJ (2007). Motivational interviewing early after acute stroke: a randomized, controlled trial. Stroke.

[8] Morreale M, Marchione P, Pili A, Lauta A, Castiglia SF, Spallone A, Pierelli F, Giacomini P (2016). Early versus delayed rehabilitation treatment in hemiplegic patients with ischemic stroke: proprioceptive or cognitive approach?. Eur J Phys Rehab Med.

[9] Kayser S, Bewernick BH, Grubert C, Hadrysiewicz BL, Axmacher N, Schlaepfer TE (2011). Antidepressant effects, of magnetic seizure therapy and electroconvulsive therapy, in treatment-resistant depression. J Psychiatr Res.

[10] Tess AV, Smetana GW (2009). Medical evaluation of patients undergoing electroconvulsive therapy. N Engl J Med.

[11] Dolenc TJ, Rasmussen KG (2005). The safety of electroconvulsive therapy and lithium in combination: a case series and review of the literature. J ECT.

[12] Yang ES, Li PW, Nilius B, Li G (2011). Ancient Chinese medicine and mechanistic evidence of acupuncture physiology. Pflugers Arch.

[13] Highfield ES, Lama P, Grodin MA, Kaptchuk TJ, Crosby SS (2012). Acupuncture and traditional Chinese medicine for survivors of torture and refugee trauma: a descriptive report. J Immigr Minor Health.

[14] Youn JI, Sung KK, Song BK, Kim M, Lee S (2013). Effects of electro-acupuncture therapy on post-stroke depression in patients with different degrees of motor function impairments: a pilot study. J Phys Ther Sci.

[15] Man SC, Hung BH, Ng RM, Yu XC, Cheung H (2014). A pilot controlled trial of a combination of dense cranial electroacupuncture stimulation and body acupuncture for post-stroke depression. BMC Complement Altern Med.

[16] Schulz KF, Altman DG, Moher D, CONSORT Group (2011). CONSORT 2010 statement: updated guidelines for reporting parallel group randomised trials. Int J Surg.

[17] MacPherson H, Altman DG, Hammerschlag R, Youping L, Taixiang W (2010). Revised STandards for Reporting Interventions in Clinical Trials of Acupuncture (STRICTA): extending the CONSORT statement. J Evid Based Med.

[18] Luisto M (1996). Guidelines for the diagnosis and treatment of patients with acute ischemic stroke [in Finnish]. Duodecim.

[19] Battle DE (2013). Diagnostic and Statistical Manual of Mental Disorders (DSM). Codas.

[20] James KE, Bloch DA, Lee KK, Kraemer HC, Fuller RK (1996). An index for assessing blindness in a multi-centre clinical trial: disulfiram for alcohol cessation—a VA cooperative study. Stat Med.

[21] Smith P, Mosscrop D, Davies S, Sloan P, Al-Ani Z (2007). The efficacy of acupuncture in the treatment of temporomandibular joint myofascial pain: a randomised controlled trial. J Dent.

[22] Whale CA, MacLaran SJ, Whale CI, Barnett M (2009). Pilot study to assess the credibility of acupuncture in acute exacerbations of chronic obstructive pulmonary disease. Acupunct Med.

[23] Chae Y, Lee H, Kim H, Sohn H, Park JH (2009). The neural substrates of verum acupuncture compared to non-penetrating placebo needle: an fMRI study. Neurosci Lett.

[24] Hamilton M (1960). A rating scale for depression. J Neurol Neurosurg Psychiatry.

[25] Sharp R (2015). The Hamilton Rating Scale for Depression. Occup Med (Lond).

[26] Zung WW (1965). A self-rating depression scale. Arch Gen Psychiatry.

[27] Chagas MH, Tumas V, Loureiro SR, Hallak JE, Trzesniak C (2010). Validity of a Brazilian version of the Zung Self-rating Depression Scale for screening of depression in patients with Parkinson’s disease. Parkinsonism Relat Disord.

[28] Brott T, Adams HP, Olinger CP, Marler JR, Barsan WG (1989). Measurements of acute cerebral infarction: a clinical examination scale. Stroke.

[29] Mahoney FI, Barthel DW (1965). Functional evaluation: the Barthel Index. Md State Med J.

[30] Zhang L, Zhong Y, Quan S, Liu Y, Shi X, Li Z, Wang J (2017). Acupuncture combined with auricular point sticking therapy for post stroke depression: a randomized controlled trial [in Chinese]. Zhongguo Zhen Jiu.

[31] Sun P, Chu H, Li P, Wang T, Pu F (2015). The effect of the acupuncture intervention of dredging Governor Vessel and regulating mentality for the medication treatment of post-stroke depression [in Chinese]. Zhongguo Zhen Jiu.

[32] Nie RR, Huang CH (2013). Post-stroke depression treated with acupuncture and moxibustion: an evaluation of therapeutic effect and safety [in Chinese]. Zhongguo Zhen Jiu.

[33] Guo RY, Su L, Liu LA, Wang CX (2009). Effects of Linggui Bafa on the therapeutic effect and quality of life in patients of post-stroke depression [in Chinese]. Zhongguo Zhen Jiu.

[34] He J, Shen PF (2007). Clinical study on the therapeutic effect of acupuncture in the treatment of post-stroke depression [in Chinese]. Zhen Ci Yan Jiu.

[35] Li HJ, Zhong BL, Fan YP, Hu HT (2011). Acupuncture for post-stroke depression: a randomized controlled trial [in Chinese]. Zhongguo Zhen Jiu.

